# *Mycobacterium tuberculosis* PE31 (*Rv3477*) Attenuates Host Cell Apoptosis and Promotes Recombinant *M. smegmatis* Intracellular Survival via Up-regulating GTPase Guanylate Binding Protein-1

**DOI:** 10.3389/fcimb.2020.00040

**Published:** 2020-02-07

**Authors:** Md Kaisar Ali, Gong Zhen, Lambert Nzungize, Andrea Stojkoska, Xiangke Duan, Chunyan Li, Wei Duan, Junqi Xu, Jianping Xie

**Affiliations:** State Key Laboratory Breeding Base of Eco-Environment and Bio-Resource of the Three Gorges Area, Key Laboratory of Eco-Environments in Three Gorges Reservoir Region, Ministry of Education, School of Life Sciences, Institute of Modern Biopharmaceuticals, Southwest University, Chongqing, China

**Keywords:** PE subfamily, cell surface, cytokines, apoptosis, guanylate-binding protein-1

## Abstract

The *Mycobacterium (M.) tuberculosis* comprising proline–glutamic acid (PE) subfamily proteins associate with virulence, pathogenesis, and host-immune modulations. While the functions of most of this family members are not yet explored. Here, we explore the functions of “PE only” subfamily member PE31 (Rv3477) in virulence and host-pathogen interactions. We have expressed the *M. tuberculosis* PE31 in non-pathogenic *Mycobacterium smegmatis* strain (Ms_PE31) and demonstrated that PE31 significantly altered the cell facet features including colony morphology and biofilm formation. PE31 expressing *M. smegmatis* showed more resistant to the low pH, diamide, H_2_O_2_ and surface stress. Moreover, Ms_PE31 showed higher intracellular survival in macrophage THP-1 cells. Ms_PE31 significantly down-regulated the production of IL-12p40 and IL-6, while up-regulates the production of IL-10 in macrophages. Ms_PE31 also induced the expression of guanylate-binding protein-1 (GBP-1) in macrophages. Further analysis demonstrates that Ms_PE31 inhibits the caspase-3 activation and reduces the macrophages apoptosis. Besides, the NF-κB signaling pathway involves the interplay between Ms_PE31 and macrophages. Collectively, our finding identified that PE31 act as a functionally relevant virulence factor of *M. tuberculosis*.

## Introduction

*Mycobacterium tuberculosis* is the main causing factor for tuberculosis (TB), leading public health concern globally (Dheda et al., 2016). According to the recent global TB report, around 6.4 million new cases of TB have appeared in the year 2017 (WHO, 2018). *M. tuberculosis* genome contains a distinctive protein family known as PE/PPE family, which contains 10% of its total genome, whose role in the virulence and pathogenesis is largely unknown. This family protein contains conserved motifs Pro-Glu (PE) and Pro-Pro-Glu (PPE) at the N-termini (Li et al., 2019). The PE family proteins hold 90–110 amino acids length of a conserved domain at N-terminal. Moreover, the PE family further classified into PE and PE_PGRS subfamilies, in the presence of GC-rich repeated sequence (PGRS) at C-terminal (Brennan and Delogu, 2002).

The bacterial cell wall is not only providing protection to the bacteria but also crucial for its pathogenesis and virulence (Abrahams and Besra, 2018). Modification in the mycobacterial cell wall components such as glycopeptidolipids usually accompanies with alteration in colony morphology and biofilm formation (Chakraborty and Kumar, 2019). Many members of PE family protein are localized and associated with the mycobacterial cell wall (Sultana et al., 2016) and secreted into the extracellular environment to interact with neighboring cells (Beatty and Russell, 2000; Beatty et al., 2001; Yu et al., 2019). The PE11 (Rastogi et al., 2017), lipY (Santucci et al., 2019), and PE_PGRS33 (Cascioferro et al., 2011), associated with the mycobacterial cell wall and PE domains of PE11 and PE_PGRS33 are responsible for translocation and localization to the cell wall (Cascioferro et al., 2007, 2011). Moreover, PE_PGRS33 (Gastelum-Avina et al., 2015), PE_PGRS41 (Deng et al., 2017), and PE11 (Singh et al., 2016) are associated with colony morphology alteration. Moreover, PE11 expressing *M. smegmatis* induced biofilm formation (Singh et al., 2016).

Most of the members of PE family protein are immunogenic and modulate the cellular processes as well as immune responses of the host, during mycobacterial infection, including macrophage immune responses, cytokines secretion, and cell death (Ahmed et al., 2015; Brennan, 2017). Invasion and survival of mycobacteria inside host macrophages is a key step for the establishment of infection. The PE_PGRS30 and PE_PGRS62 are vital for intracellular survival of mycobacteria in macrophages (Ahmed et al., 2015). PE_PGRS33 interact with TLR2 and activate the macrophages to release the cytokines and modulate the host cell apoptosis (Basu et al., 2007; Palucci et al., 2016). Moreover, PE9–PE10 protein pairs interact with macrophage TLR4 to induce the apoptosis and modulate of cytokine secretion (Tiwari et al., 2012).

GBP-1 is an interferon-stimulated gene belonging to the GTPase family and expressed in several cell types including macrophages (Guenzi et al., 2001) and up-regulated in inflammatory tissues (Degrandi et al., 2007; Kim et al., 2011; Pilla-Moffett et al., 2016). The siRNA silenced GBP-1 cells become favorable toward the apoptosis, accompanied by more pro-inflammatory cytokines secretion (Schnoor et al., 2009). Many bacterial pathogens target the GBPs and manipulate it in cell-specific manners (Ngo and Man, 2017). Mycobacteria effectors interfere with several signaling pathways, including NF-κB to regulate the downstream cytokines, inflammatory molecules (Naschberger et al., 2004; Cao et al., 2006), and several proteins associate with apoptosis (Voboril and Weberova-Voborilova, 2007).

Our interest focused on *Rv3477* gene encoded PE31 protein. Previously, the PE31 interact with PPE18, and able to form a heterodimeric complex with TLR 2 (Mukhopadhyay and Balaji, 2011). While another report suggested that PE31 with PE51 are protective antigens (Myllymaki et al., 2017). However, the exact role of PE31 in pathogenicity, host-pathogen interaction and underlying mechanisms are unknown. We found that PE31 plays an influential role in the alteration of colony morphology and biofilm formation. In addition, PE31 enhanced the *M. smegmatis* resistance to the stresses such as, low pH, nitrogen intermediate, reactive oxygen species and surface stress, and boosted its survival within macrophages. Moreover, PE31 altered the macrophage secretion profile, GBP-1 protein expression and reduced macrophages apoptosis, by activating the NF-κB signaling.

## Materials and Methods

### Bacteria, Growth Environments, and Cell Culture

For gene transformation and cloning, we used *Escherichia coli* DH5α strain, cultured with pertinent antibiotics in LB medium at 37°C. *M. smegmatis* mc^2^155 replicated in Middlebrook 7H9 liquid or Middlebrook 7H10 medium contained glycerol (0.5%, v/v), glucose (0.2%, w/v) and Tween 80 (Tw) (0.05%, v/v). When needed, 100 μg/ml of hygromycin (Hyg) supplied in the medium. All strains were preserved in −70°C mixed with 20% (v/v) glycerol, for further use.

The RPMI 1640 added fetal bovine serum (10%) medium was used to seed the THP-1 human macrophage cells, supplemented 100 U/ml of penicillin (Pen), 100 μg/ml of streptomycin (Str) and L-glutamine (Glu) (2mM) (GIBCO, Invitrogen), incubated in 5% CO_2_ containing atmosphere at 37°C.

### The Construction of PE31 Recombinant *M. smegmatis*

The pALACE expression vector was used for the construction of recombinants. For amplification of PE31 gene from *M. tuberculosis* H37Rv genome, specific primers were used (**Table 2**). To construct the pALACE- PE31, amplified PCR yield was digested by restriction enzymes *Cla*I and *Bam*HI and cloned into the pALACE. Then, electroporation applied to incorporate the plasmids (pALACE and pALACE-PE31) into the *M. smegmatis* mc^2^155. The recombinant *M. smegmatis* mc^2^155 was picked on Middlebrook 7H9 solid medium supplied with 100 μg/ml of Hyg. The PE31 gene containing recombinant strains were confirmed by PCR amplification. Strains and plasmid used in this study are mentioned in Table 1.

**Table 1:** Used strains and plasmids.

**Strains**	**Description**
*Mycobacterium smegmatis* mc^2^ 155	ATCC 700084 isolation, efficient plasmid transformation to the characterization of mutant *M. smegmatis*
*Escherichia coli DH5α*	Used in vector multiplication that transformed to *M. smegmatis*
pALACE	A replicative plasmid used for expression of gene of interest in *M. smegmatis*, conferring by hygromycin resistance
Ms_PE31	*M. smegmatis* transformed with vector pALACE_PE31
Ms_vec	*M. smegmatis* transformed with vector pALACE

### Heterogeneous Expression of PE31 in Recombinant *M. smegmatis*

Ms_PE31 and Ms_vec, cultured in 100 μg/ml Hyg supplemented Middlebrook 7H9 liquid medium. For protein expression, when OD_600_ reached 0.6, 28 mM of acetamide (Ace) was added (Aladdin, China). In detail, after 16 h of Ace induction, both recombinant strains were collected by centrifugation at speed 3,000 × *g* 10 min at 4°C. Then, wash the harvested cells with 1 × PBS and sonicated in ice-cold PBS. Later, it centrifuged at speed 20,000 × *g* and collected the whole cell lysate to separate into soluble (supernatant in the upper layer) and insoluble (bottom pellets) fractions. Western blot employed to detect the SDS-PAGE separated sediments by adopting anti-His monoclonal antibody, and secondary antibody IgG-HRP, a horseradish peroxidase-labeled anti-mouse IgG monoclonal antibody (TIANGEN, China).

### Growth Kinetics Assay

Recombinant bacterial strains growth kinetics accomplished in Tw (0.05%, v/v) added Middlebrook 7H9 broth liquid medium. Starting bacterial growth of both strains (Ms_PE31 and Ms_vec) were equalized at OD_600_ 0.03, and cultured at 37°C with continuous shaking. When OD_600_ reached 0.8, inducer (Ace) was added and monitored the OD_600_ for every 4 h up to 72 h. The growth curve was plotted between OD_600_ vs. time intervals.

### *In-vitro* Survival Under Different Stress Conditions

To perform acidic stress, the pH gradient of the Middlebrook 7H9 liquid medium was maintained by adding hydrochloric acid. The recombinant bacterial strains (Ms_PE31 and Ms_vec) were treated with acidic exposure, for 0, 3, 6, and 9 h time points. Then, took 100 μl at mentioned time points and dappled onto Middlebrook 7H9 solid media plates containing Hyg by 10-fold serial dilution. Three days post-incubation, CFUs were computed.

To measure the effect of H_2_O_2_ and SDS, disc diffusion method was performed. The mid-exponential-phase of recombinant strains (Ms_PE31 and Ms_vec) was used for this experiment. Briefly, 10 μl SDS of concentrations 2.5, 1.25, and 0.625%, and 10 μl H_2_O_2_ of concentrations 0.5, 1, and 2% (v/v) were dropped on the Whatman filter disc of 5.5 mm-diameter on the bacterial lawn.

For diamide stress, spot test was performed. When recombinant bacterial strains (Ms_PE31 and Ms_vec) OD_600_ reached 0.8, 10-fold serial diluted samples were dappled on the Middlebrook 7H9 solid plates supplemented with indicated diamide concentrations. After 3 days of incubation, surface layer was detected.

### Cell Surface Characteristics Analysis

For analysis of cell morphology, recombinant strains of bacteria (Ms_PE31 and Ms_vec) were cultured. When OD_600_ reached 1, cultured bacteria were collected and washed. After that washed bacteria were re-suspended into sterile Middlebrook 7H9 broth containing 2% (w/v) Ace and incubated in an incubator at 37°C for 3 days. The colony size and surface wrinkles were recorded.

For the biofilm formation assay, the recombinant bacterial strains were cultured. When OD_600_ reached 1, cultured bacteria were harvested and re-suspended in sterile Middlebrook 7H9 broth. Cells were put into Middlebrook 7H9 liquid medium containing 6-well polystyrene plate, without Tw. The plate was shifted to 37°C without shaking. After 3 days of incubation, layer of the surface was detected. Tetrahydrofuran (THF) assay was carried out for the biofilm quantification as described previously (Syal, 2017) with minor modifications. Briefly, the media was discarded from the wells and underneath the biofilm at the interface through a syringe. After that, biofilms were washed twice applying deionized water and dried it. Subsequently, absorbance recorded at 595 nm, of biofilm suspended THF (200 μl).

### Macrophages Infection

The RPMI 1,640 added fetal bovine serum (10%) medium was used to seed the macrophage THP-1 cells, supplemented with 100 U/ml of Pen, 100 μg/ml of Str and Glu (2 mM) (GIBCO, Invitrogen) and incubated in 5% CO_2_ containing atmosphere at 37°C. 1 × 10^6^ cells each well were germinated in 12-well and 24-well culture plates. Differentiation of cells was induced pre-infection by adding Phorbol 12-myristate 13-acetate (PMA), 100 ng/ml (Sigma). Then, infection of differentiated cells was performed by recombinant bacteria at MOI = 10. After 4 h post-infection, infected cells were washed by applying PBS and 100 μg/ml gentamicin supplemented to eliminate the bacteria. At 6, 24, 48, and 72 h post-infection, sterilized PBS applied to wash the cells triplet and lysed by adding SDS (0.025%, w/v) to emancipate the intracellular bacteria. 10-fold serial diluted lysed cells were mottled on the Middlebrook 7H9 solid media containing appropriate antibiotics. The bacterial number was inventoried after 3 days of incubation.

### Cytokines Production Assay

Recombinant strains Ms_PE31 and Ms_vec were infected to the PMA-differentiated THP-1 cells at MOI = 10. Total RNAs were collected at post-infection, by RNA isolation kit (TIANGEN), as per manufacturer's protocol. The cDNA synthesis was employed according to the manufacturer's guidance (TIANGEN, China). qRT–PCR employed to detect the relative levels of mRNA expression, while β-actin used as the internal control. Concerted gene primers are mentioned in Table 2. Culture supernatants were harvested and the level of cytokines determined using ELISA kits, following manufacturer's protocols (eBioscience).

**Table 2:** Used primers.

**Primers**	**Sequence (5**′**−3**′**)**
pALACE-PE31-F	CGGGATCCAAATGAGGAGGAGCACGCGTGTCTT
pALACE-PE31-R	CCATCGATCGAATACCGTCAGGTCAGCTAGCCG
IL-6-F	GCCTTCGGTCCAGTTGCCTTCT
IL-6-R	TGCCAGTGCCTCTTTGCTGCTTT
IL-10-F	ACCTGGGTTGCCAAGCCTTGT
IL-10-R	GCTCCACGGCCTTGCTCTTGTTT
IL-12p40-F	CATCATCAAACCTGACCCACC
IL-12p40-R	CTTTTCTCTCTTGCTCTTGCCC
hGBP1-F	CGAGGGTCTGGGAGATGTAG
hGBP1-R	TAGCCTGCTGGTTGATGGTT
β-actin-F	GTGACGTTGACATCCGTAAAGA
β-actin-R	TGTGAGTCCCGGAGCGTGCAGTT

### Apoptosis Analysis

Recombinant strains (Ms_PE31 and Ms_vec) have infected to 2 × 10^6^ THP-1 cells. At 24 h post-infection, PBS applied to wash the cells 3 times and added the annexin V-FITC and propidium iodide (PI) stains as followed by the manufacturer's instructions (Beibo, Shanghai, China). Analysis of apoptosis was employed by flow cytometer and fluorescence microscopy. For negative control, untreated cells were used.

### Western Blotting

Recombinant strains (Ms_PE31 and Ms_vec) were infected to the THP-1 cells. At 24 and 48 h post-infections, PBS applied to wash the cells. After that, mammalian cell lysis buffer (Sigma) added to lyse the cells and centrifuged at 12,000 × g for 15 min. The protein concentrations were quantified by using BCA method (TIANGEN, China). After that, SDS-PAGE was employed to disassociate the same quantities cell lysates, then shifted on the nitrocellulose membrane. Dry milk (5%, w/v) supplemented TBST was applied for blocking the membranes, then incubate in relevant primary antibodies against caspase-3, cleaved caspase-3 and hGBP-1 (dilution 1: 1,000) for overnight. After that, membranes were shifted for 1 h in specific HRP-tagged secondary antibodies and obtained the X-ray film to detect the protein expression levels by using Plus-ECL chemiluminescent reagent. β-actin was considered as an internal control. Densitometry analysis of the images was performed by ImageJ software.

### Signaling Pathway Inhibition

TPCK (tosyl phenylalanyl chloromethyl ketone) used as a specific inhibitor of NF-κB signaling pathway (Gong et al., 2019). TPCK (30 mM) pre-treated for 1 h to the PMA- induced THP-1 cells, and DMSO (0.1%, v/v) pre-treated cells were considered as a control group. At 24 h post-infection, total RNAs were collected by RNA isolation kit (TIANGEN), as per manufacturer's protocol, and converted into the cDNA according to the manufacturer's guidance (TIANGEN, China), by using following parameters: 37°C/15 min → 85°C/5 s.

### Statistical Analysis

Experiments were employed independently triplicates. GraphPad Prism 6 employed to determine group differences. Student's *t*-test applied to compute the *p*-value. ^*^P < 0.05, ^**^P < 0.01 and ^***^P < 0.001 values were calculated from triplicates. The error bars represent standard deviation of the mean.

## Results

### PE31 Expressing *M. smegmatis* Altered the Cell Surface Characteristics

**Figure 1:**
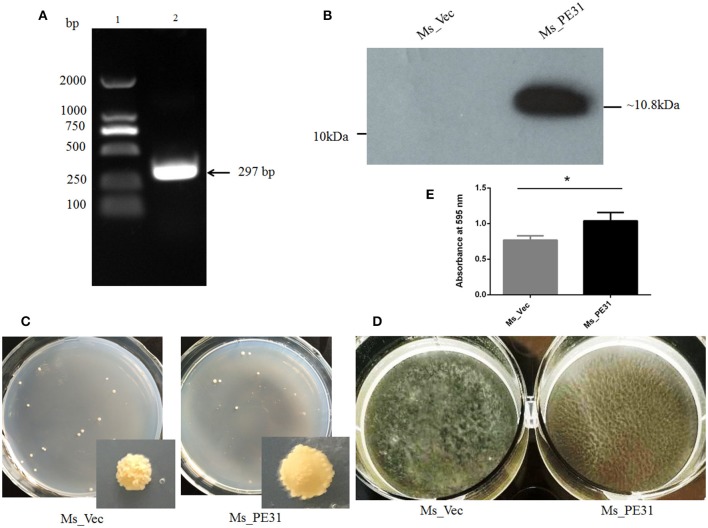
Ms_PE31 can alter the surface characteristics of *M. smegmatis*. **(A)** PE31 (Rv3477) sequence amplified by PCR, applying *M. tuberculosis* genome, ~297 bp in length (lane 1 = DNA ladder and lane 2 = amplified gene). **(B)** The Ace-induced recombinant cell lysates were prepared and employed the Western blot for confirmation of His-tagged PE31 protein expression. **(C)** Both recombinant strains cultured on plates containing 7H9 solid media with hyg and Ace (0.02%, w/v), the developed single colony **(D)** Recombinant strains cultured in Ace (0.02%, w/v) added 7H9 liquid on polystyrene plates to induce the development of biofilm. **(E)** The quantification of biofilm formation was confirmed by Tetrahydrofuran (THF) assay (*n* = 3). Results were determined by Student's *t*-test, ^*^P < 0.05. Error bars represent the standard deviation of mean.

To scrutinize the role of PE31, *M. smegmatis* fast-growing surrogate host was used. PCR amplified PE31 (*Rv3477*) gene (297 bp) (Figure 1A) were used to construct the recombinant strain. The recombinant strain Ms_PE31 contained His-tagged pALACE_PE31 vector, while pALACE only vector containing Ms_vec strain served as a control. The recombinant strains were cultured in Hyg added 7H9 medium. PE31His-tagged protein expression in Ms_PE31 was validated by western blotting, while absent in Ms_vec (Figure 1B).

To explore whether PE31 alters the cell surface characteristics, the colony morphology and biofilm developing capability of the Ms_PE31 and Ms_vec were measured. There was a dramatic transformation observed in colony morphology, growth in 7H9 solid media. Ms_vec showed the frill, bulging and rough colony, while Ms_PE31 colony surface was sleeker and damp (Figure 1C). Moreover, Ms_PE31 produced the more massive biofilm, as compared to Ms_vec produced biofilm on the Middlebrook 7H9 liquid medium containing hydrophobic polystyrene surface (Figure 1D). The biofilm quantified by the tetrahydrofuran (THF) assay, validated this result (Figure 1E). These results implicated the role of PE31 in transforming the cell surface properties of *M. smegmatis*.

### PE31 Foster the *M. smegmatis* Resistance to Stress

**Figure 2:**
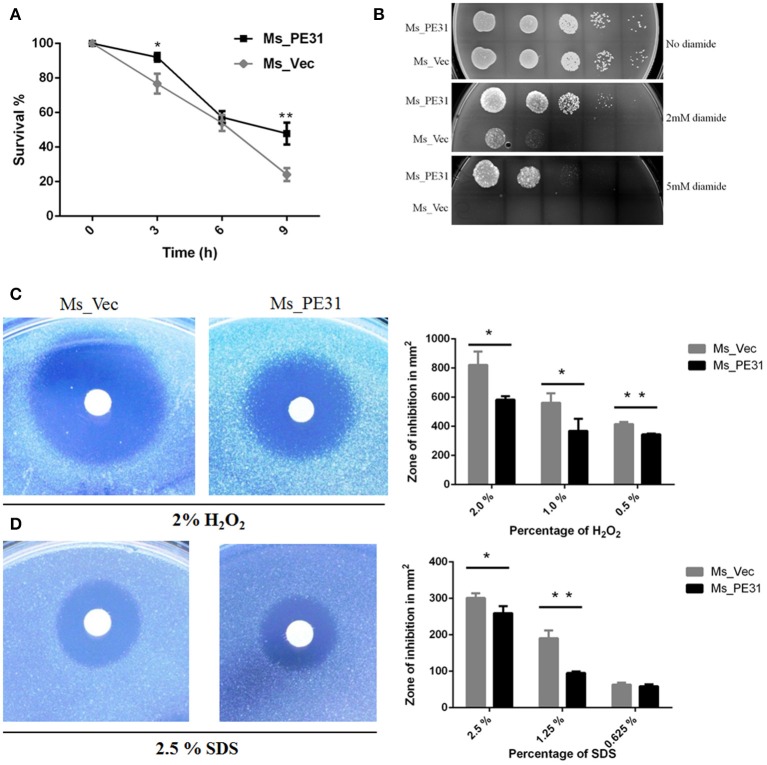
Ms_PE31 increase growth under multiple stress. **(A)** The growth rate of recombinant strains was measured under *in-vitro* low pH condition. Cultured bacteria were harvested and re-cultured in 7H9 (pH = 5) to maintained the OD_600_ =0.8. Then, incubated in 37°C and 100 μl taken from it after different time intervals for workable enumeration (*n* = 3). **(B)** The reactive nitrogen stress was examined by spot test, the supplementation of 2 and 5 mM diamide to the 7H9 solid medium to grown the Ms_Vec and Ms_PE31 strains. Growth of bacteria on 7H9 solid media which contained mentioned concentrations of diamide (*n* = 3). **(C,D)** Ms_Ves and Ms_PE31 survival upon exposure to H2O2 and SDS, respectively, examined by disk diffusion technique. Whatman disks used to mottled the different concentrations of H2O2 (10 μl) and SDS (10 μl) (*n* = 3). Area of the zone of inhibition was calculated after 3−4 days incubation at 37°C. The results were determined by Student's *t*-test, ^*^P < 0.05 and ^**^P < 0.01. Error bars represent the standard deviation of mean.

Upon invasion of *M. tuberculosis* within macrophages exposed with stressful environment, including low pH, reactive oxygen species (), and reactive nitrogen intermediates (RNIs) (Zhai et al., 2019). To explore, whether PE31 confer the resistance to multiple-stress, the growth pattern between Ms_PE31 and Ms_vec were compared upon exposed with low pH, RNIs, and. We observed that Ms_PE31 survival percentage was higher as compared with Ms_vec after treated with the acid environment (pH 5) (Figure 2A). Though, 7H9 media containing 0, 2, and 5 mM diamide was used to mimic the potency of RNIs. We found that Ms_PE31 showed significantly more survival as compared to Ms_vec upon exposed with 2 and 5 mM diamide (Figure 2B). For reactive oxygen defiance, the zone of inhibition was measured. Upon exposed with 0.5, 1, and 2% (v/v) H_2_O_2_, Ms_PE31 showed the smaller zone of inhibition, as compared to Ms_vec (Figure 2C), indicate that Ms_PE31 was significantly more resisted to the H_2_O_2_ as compare to Ms_vec. Similarly, the zone of inhibition was measured, upon treated with 2.5, 1.25, and 0.625% (w/v), which mimic the surface stress. Ms_PE31 showed the smaller zone of inhibition than Ms_vec (Figure 2D), indicating that Ms_PE31 was more resisted to the SDS, with the comparison of Ms_vec. Collectively, these results demonstrated that Ms_PE31 can bestow a competitive reward under stresses which might *M. tuberculosis* encounter within the host macrophages.

### Ms_PE31 Enhances Intracellular Survival in Macrophages

**Figure 3:**
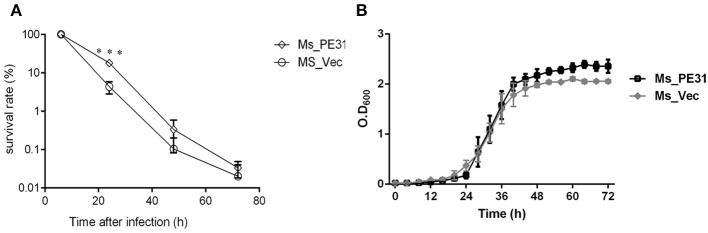
Survival rate of *M. smegmatis* recombinant strains. **(A)** Recombinant strains infected macrophages were laved and lysed SDS (0.025%, w/v) at indicated intervals. 10-fold serial diluted lysed cells were mottled on hyg containing 7H9 solid medium plates. After 3–4 days, CFU was computed (*n* = 3). **(B)** Growth kinetics of recombinant strains were determined by the growth of bacteria in 7H9 liquid added Ace (1%, w/v), Tw (0.05%, v/v) and hyg (100 μg/ml) (*n* = 3). Results were determined by Student's *t*-test, ^***^P < 0.001. Error bars represent the standard deviation of mean.

The interactions and survival of *M. tuberculosis* within macrophages is the primarily stage of infection. To scrutinize, whether PE31 enhance the survival of *M. smegmatis* within the host macrophages, the THP-1 cells were infected with Ms_PE31 and Ms_vec at MOI = 10. At indicated time points post-infection, the intracellular bacteria were assessed in THP-1 cells and calculating the survival percentage. The result showed that bacilli recovered from Ms_PE31 infected THP-1 cells were significantly higher as compared to Ms_vec infected THP-1 cells, at 24 h of infection, while significant difference was not observed in further time-points (Figure 3A). However, no notable difference was observed in the *in-vitro* growth of Ms_PE31 and Ms_vec (Figure 3B). These data suggested that PE31 promoted to the intracellular continuance of *M. smegmatis* within macrophages.

### Macrophage Cytokines Profile Was Changed by Ms_PE31

**Figure 4:**
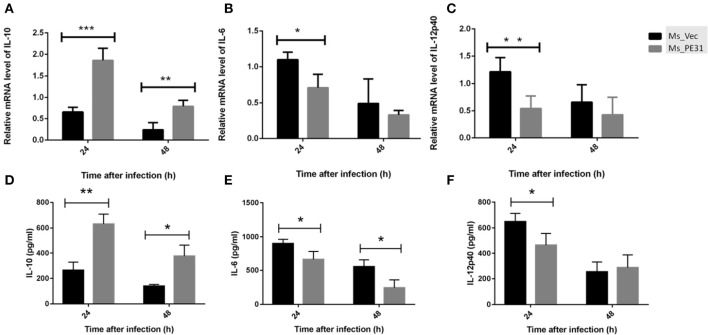
*M. tuberculosis* PE31 expressed recombinant *M. smegmatis* regulated the cytokines profile. Ms_PE31 and Ms_Vec infected differentiated human macrophages, total RNA collected to carried out qRT-PCR and analyzed the relative mRNA of **(A)** IL-10 (*n* = 4), **(B)** IL-6 (*n* = 4), **(C)** and IL-12p40 (*n* = 4). For all RT-PCRs, β-actin of macrophages for internal control. Infected cells supernatant collected, and ELISA was accomplished to detect the production of **(D)** IL-10 (*n* = 3) **(E)** IL-6 (*n* = 3) **(F)** and IL-12p40 (*n* = 3). Results were determined by Student's *t*-test, ^*^P < 0.05, ^**^P < 0.01, and ^***^P < 0.001. Error bars represent the standard deviation of mean.

Cytokines are pivotal participants in the balance of pathogen-host interplay (Zhai et al., 2018). To explore the effect of PE31 on macrophages secreted cytokines modulations, PMA-induced THP-1 cells were infected with Ms_PE31 and Ms_vec. After 24 and 48 h post-infection, total RNAs were extracted and performed the RT-PCR to analyze the transcriptional level of cytokines, by using specific primers (Table 2). The supernatant was also harvested from the same sample and measured the translational level of cytokines, by using ELISA kits. The result showed that the transcriptional and translational level of IL-10 was significantly increased (Figures 4A,D) and IL-6 level was significantly decreased (Figures 4B,E) in Ms_PE31 infected THP-1 cells, as compared to Ms_vec infected cells, at 24 and 48 h post-infection. While the transcriptional and translational level of IL-12p40 (Figures 4C,F) was significantly decreased in Ms_PE31 infected THP-1 cells than Ms_vec infected THP-1 cells, at 24 h post-infection. These data suggested that Ms_PE31 regulated the cytokines profile of macrophages.

### Ms_PE31 Attenuates the Macrophage Apoptosis

**Figure 5:**
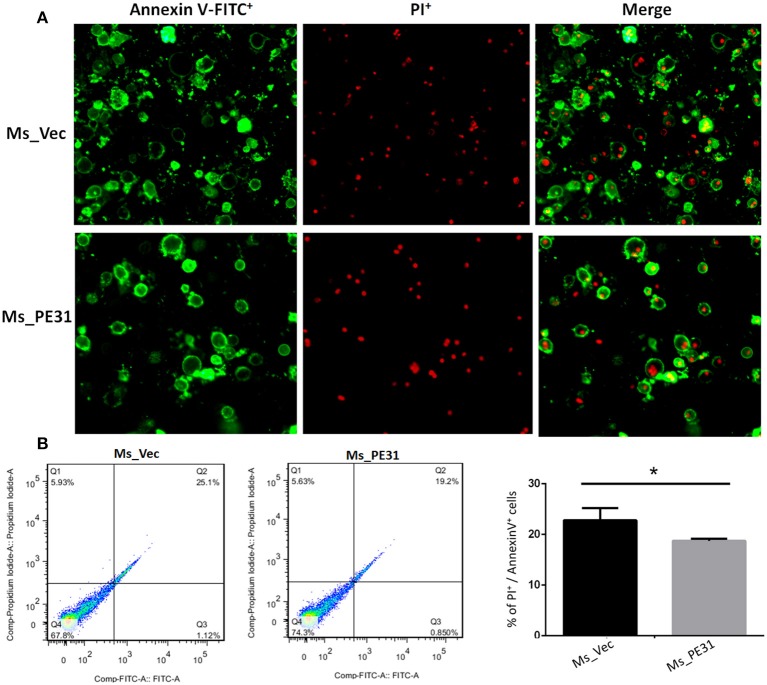
Ms_PE31 reduced the cell apoptosis of macrophage. Recombinant strains infected PMA differentiated macrophages were stained by Annexin V-FITC/ PI, and collected to measure the apoptosis levels by using **(A)** fluorescence microscope **(B)** flow cytometry (*n* = 3). Results were determined by Student's *t*-test, ^*^P < 0.001. Error bars represent the standard deviation of mean.

**Figure 6:**
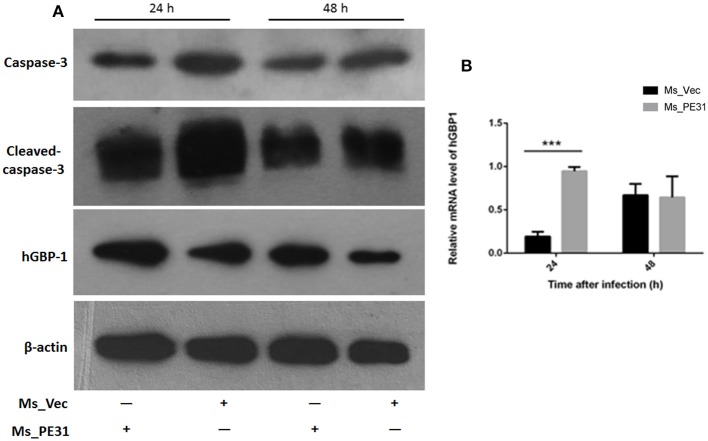
Ms_PE31 limits the apoptosis of macrophage via GBP-1 dependent manner. Recombinant strains infected PMA differentiated macrophages at post-infection **(A)** washed and detected the expression of caspase-3, activated caspase-3 and hGBP-1 proteins, by western blot. **(B)** qRT-PCR was executed, by using isolated RNA, to analyzed mRNA level of hGBP-1 (*n* = 3). In both experiments, β-actin considered as internal control. Results were determined by Student's *t*-test, ^***^P < 0.001. Error bars represent the standard deviation of mean.

The secreted cytokines in macrophages tend to trigger the apoptosis (Liu et al., 2015). To test the fate of PE31 on apoptosis, recombinant strains (Ms_PE31 and Ms_vec) infected PMA-differentiated THP-1 cells. After 24 h post-infection, infected THP-1 cells were stained with annexin-V together with PI to detect outer leaflet presented phosphatidylserine of apoptotic cells, using fluorescence microscopy and flow cytometry. We observed in the case of Ms_vec infected THP-1 cells, apoptosis level of Ms_PE31 infected THP-1 cells were significantly diminished (Figure 5A). This is also confirmed by the flow cytometry data (Figure 5B). The western blot result showed that expression level of caspase-3 and activated caspase-3 proteins were diminished in Ms_PE31 infected THP-1 cells than Ms_vec infected THP-1 cells (Figure 6A and Supplementary Figure 1). This data suggested that PE31 is able to reduce macrophages apoptosis.

### Ms_PE31 Induced the GBP-1 in Macrophage

GBP-1 is an inflammatory molecule and usually up-regulated during bacterial infection, inflammation outcomes and associated with apoptosis (Mirpuri et al., 2010). To affirm, whether PE31 induce the GBP-1 protein, transcriptional level, and protein expression were analyzed from Ms_PE31 and Ms_vec infected THP-1 cells. After 24 and 48 h post-infection, RNAs were extracted to perform the RT-PCR by using hGBP-1 specific primer (Table 2), and cells lysate were extracted to perform the western blot. Our result showed that the transcriptional level of hGBP-1 was significantly higher in Ms_PE31 infected THP-1 cells, compared to Ms_vec infected cells, at 24 h time-point (Figure 6B). The western blot result of hGBP-1 protein expression in Ms_PE31 and Ms_vec infected THP1 cells also supported the above result (Figure 6A and Supplementary Figure 1). These results suggested that Ms_PE31 unregulated the GBP-1 protein in macrophages.

### Ms_PE31 Regulates the Inflammatory Molecules via NF-κB Pathway

**Figure 7:**
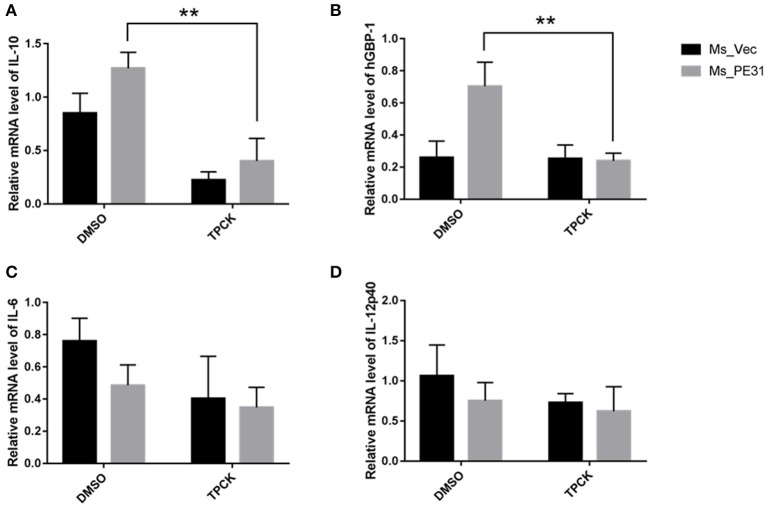
Ms_PE31 regulates the inflammatory molecules in macrophages via NF-κB signaling. TPCK (NF-κB inhibitor) pre-treated differentiated THP-1 cells were infected with recombinant strains. Total RNA was collected to carry out qRT-PCR to detect the mRNA level of **(A)** IL-10 (*n* = 3) **(B)** hGBP-1 (*n* = 3) **(C)** IL-6 (*n* = 3) **(D)** and IL-12p40 (*n* = 3). The β-actin gene of macrophages cells was used for internal control. Results were determined by Student's *t*-test, ^**^P < 0.01. Error bars represent the standard deviation of mean.

To investigate the underlining mechanism how PE31 activate NF-κB signaling to regulate the secretion of above mentioned cytokines and GBP-1. We treated the PMA-differentiated THP-1 cells by specific NF-κB inhibitor (TPCK) (Gong et al., 2019) before 1 h of infection with Ms_PE31 and Ms_vec. After 24 h post-infection, cells were collected, lysed and RNAs were isolated. The transcriptional level cytokines and inflammatory molecules were analyzed by RT-PCR, using specific primers (Table 2). We found that after treatment with TPCK, the transcriptional level of IL-10 (Figure 7A) and GBP-1 (Figure 7B) was significantly declined in Ms_PE31 infected THP-1 cells, as compared to Ms_vec infected THP-1 cells. While no obvious difference was observed in IL-6 (Figure 7C) and IL-12p40 (Figure 7D) levels after treated with TPCK in both recombinant strains (Ms_PE31 and Ms_vec) infected THP-1 cells. Taken together, these data suggested that Ms_PE31 activated the NF-κB signaling pathway to mediate the expression of cytokine IL-10 and GBP-1 protein in macrophages.

## Discussion

Over the past decade, accumulating evidence suggested that PE subfamily proteins are actively involved in the virulence, antigenic variation, and immune modulations in the host during *M. tuberculosis* infection (Brennan, 2017; Li et al., 2019). The involvement of “PE only” subfamily in these processes has largely unknown.

In this study, we investigate the function of PE31, a member of PE subfamily, has not been studied previously. Few members of this subfamily associated with cell wall and involved in the alteration of cell surface properties (Singh et al., 2016; Rastogi et al., 2017). The colony morphology in mycobacteria is a complex structure and associate with the virulence, cytokines production as well as signaling activation (Singh et al., 2016). Previous study suggested that the alteration in colony morphology correlated with virulence and metabolic changes in *M. avium* (Kansal et al., 1998). We found that PE31 gene expressing *M. smegmatis* altered the colony morphology, shifted from usual coarse and dry to unusual curious sleeker and damp, implicating the involvement of structural role and virulence of this protein (Maya-Hoyos et al., 2015). Biofilms facilitate the mycobacteria to become tenacious in the host (Chen et al., 2006) and undergird the aptness of its infection. We observed that *M. smegmatis* expressing PE31 gene induced the biofilm formation. However, further study needs to investigate the differences in cell envelop lipids and glycolipids component.

As previously, reported vaccine candidate (Myllymaki et al., 2017), the role of PE31 in host interactions and pathogenesis of *M. tuberculosis* has remained to be understood. *M. tuberculosis* survival within macrophages is necessitates effective neutralization of hostile environments such as acidification, oxygen radicals, and RNIs (Cossu et al., 2012; Li et al., 2017). We found that PE31 expressing *M. smegmatis* became more resisted toward low pH, H_2_O_2_, diamide, and SDS as compared to control evidenced that Ms_PE31 able to survive in the stressful environment inside host macrophages might be leads to initiate the infection. This evidence was supported by the increased intracellular survival rate of Ms_PE31 observed in macrophages.

Cytokines are main player in interplay between the *M. tuberculosis* and host. IL-10 (O'Leary et al., 2011), IL-6 (Ponnana et al., 2017), as well as IL-12p40 (Cooper and Khader, 2007) are essential for the host immune counter against mycobacteria (Hossain and Norazmi, 2013). The interplay between IL-10, IL-12p40, and IL-6 intense affected the outcomes of macrophage function and bacterial infection (Hussain et al., 2016). The neutralization of IL-6 boosted the *M. tuberculosis* survival in T2DM mice (Cheekatla et al., 2016). The human with deficient IL-12p40 is more susceptible to mycobacterial infection (Cooper and Khader, 2008). IL-10 is not only crucial immune-regulatory molecule, but also able to inhibit the anti-mycobacterial activity of macrophages (Nagata et al., 2010) including, blocking phagosomal maturation and apoptosis, and decreasing the production of pro-inflammatory cytokines, accounts for the intracellular survival of mycobacteria (Hussain et al., 2016). Consistently, we found that Ms_PE31 infected macrophages increased the production of anti-inflammatory cytokine IL-10, while decreased the production of specific pro-inflammatory cytokines IL-6 and IL-12p40, might be promoted to the survival of Ms_PE31 in macrophages and induces the other immune responses.

At the early stage of infection, apoptosis is a considerable host defenses tackles of macrophages to obliterate the *M. tuberculosis* (Liu et al., 2015). Thus, the *M. tuberculosis* inhibits the apoptosis and leads to escape the host immunity and latent infection (Zhai et al., 2019). We detected that annexin V-FITC^+^/PI^+^ cells percentage in Ms_PE31 infected macrophages were significantly lower compared to control at the early stage of infection, indicating that Ms_PE31 reduced the apoptosis of the macrophages. Activation of caspase-3 protein is the critical step for the execution of apoptosis (Choudhary et al., 2015). We found that activated caspase-3 was decreased at early stage of infection in Ms_PE31 infected macrophages in compared with control, supported the evidence of reduced apoptosis of macrophages at the early stage of infection.

The GBP-1 is 65 kD GTPase protein plays a crucial role in innate immunity (Qiu et al., 2018) as well as a prognostic biomarker for infection outcome (Degrandi et al., 2007; Kim et al., 2011; Pilla-Moffett et al., 2016). Previous studies suggested that during active inflammation in the intestinal epithelial cells, GBP-1 showed to be up-regulated and influence the several cellular processes, including modulation of cytokines, caspases, and prevention of apoptosis (Capaldo et al., 2012; Qiu et al., 2018). Our result found that Ms_PE31 infected macrophages induced GBP-1 protein expression might be involved in the attenuation of macrophages apoptosis and other immune responses. But, the actual role of this GBP-1 in *M. tuberculosis* infection remains to be defined.

*M. tuberculosis* can activate the NF-κB signaling pathway to inhibit the host cells apoptosis (Wang et al., 2014), by regulating the IL-10 (Cao et al., 2006) and GBP-1 (Naschberger et al., 2004). We demonstrate that the occlusion of NF-κB by specific inhibitor in Ms_PE31 infected macrophages leads to down-regulated the IL-10 and GBP-1 level, with unknown underlying mechanisms. Further investigations required to confirm the underlying pathways in the expression of such cytokines and inflammatory molecules.

**Figure 8:**
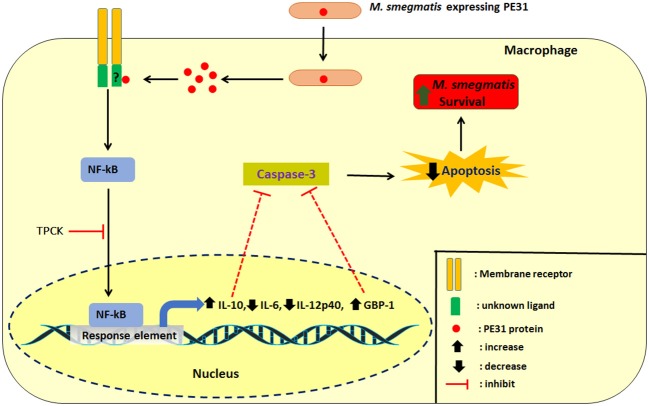
Schematic delineation of Ms_PE31 and interaction with macrophage proteins. Ms_PE31 induced the IL-10, GTPase family protein GBP-1 and down-regulates the IL-12p40 and IL-6 feasibly by the NF-κB pathway. These proteins inhibit the activation of caspase-3 to reduce the macrophages apoptosis level and boost the recombinant *M. smegmatis* survivorship in macrophages.

In summary, we demonstrated that intracellular Ms_PE31 in macrophages might be secreting the PE31 protein, where it interacts with unknown ligand to activate the NF-κB signaling pathway. The NF-κB downstream signaling induced the IL-10 and GBP-1 might be inhibited the activation of caspase-3 and leads to attenuate the macrophages apoptosis, thereby fostering its intracellular survival of mycobacteria and establishment of infection (Figure 8).

## Data Availability Statement

The raw data supporting the conclusions of this article will be made available by the authors, without undue reservation, to any qualified researcher.

## Author Contributions

MA designed experiments, data analysis, and wrote the manuscript. MA performed all experiments with GZ contributing to intracellular survival assay, cytokines assay, and apoptosis assay. CL performed the *in-vitro* stress assay. WD performed statistical analysis. JXu performed the cell surface characteristics assay. LN, AS, and XD edited the manuscript. JXi reviewed the manuscript and supervised the research. All authors read and approved the manuscript.

### Conflict of Interest

The authors declare that the research was conducted in the absence of any commercial or financial relationships that could be construed as a potential conflict of interest.

## Supplementary Material

Supplementary Figure 1 Densitometry analysis of western blot images. The densitometry analysis of obtained western blot images (caspase-3, cleaved caspase-3, hGBP-1, and β-actin proteins) were performed by using ImageJ software, and determined the area percent of each band.
