# Supplementary figures and images for: Mycobacterium tuberculosis PE31 (Rv3477) Attenuates Host Cell Apoptosis and Promotes Recombinant M. smegmatis Intracellular Survival via Up-regulating GTPase Guanylate Binding Protein-1
Source: Front Cell Infect Microbiol. 2020 Feb 7;10:40. doi: 10.3389/fcimb.2020.00040 (PMC7020884; doi:10.3389/fcimb.2020.00040)

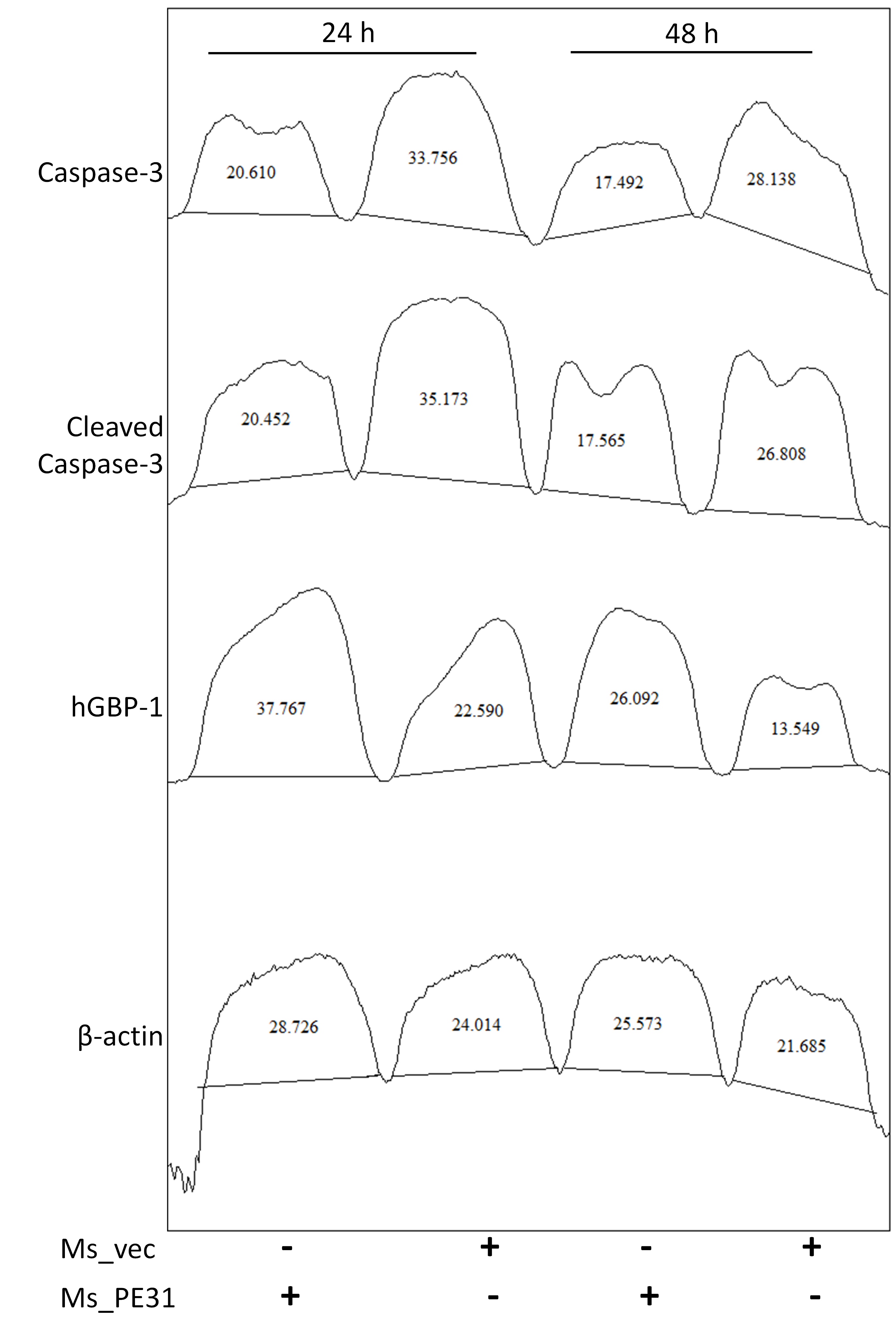

Supplement: Supplementary Figure 1 — Densitometry analysis of western blot images. The densitometry analysis of obtained western blot images (caspase-3, cleaved caspase-3, hGBP-1, and β-actin proteins) were performed by using ImageJ software, and determined the area percent of each band. [file Image_1.TIF]
